# Biological Planning of Radiation Dose Based on In Vivo Dosimetry for Postoperative Vaginal-Cuff HDR Interventional Radiotherapy (Brachytherapy)

**DOI:** 10.3390/biomedicines9111629

**Published:** 2021-11-06

**Authors:** Tamer Soror, Ramin Chafii, Valentina Lancellotta, Luca Tagliaferri, György Kovács

**Affiliations:** 1 Radiation Oncology Department, University of Lübeck/UKSH-CL, 23562 Lübeck, Germany; r.chafii@gmail.com; 2 National Cancer Institute (NCI), Radiation Oncology Department, Cairo University, Giza 12613, Egypt; 3 UOC Radioterapia Oncologica, Dipartimento di Diagnostica per Immagini, Radioterapia Oncologica ed Ematologia, Fondazione Policlinico Universitario A. Gemelli, IRCCS, 00168 Roma, Italy; valentina.lancellotta@policlinicogemelli.it (V.L.); luca.tagliaferri@policlinicogemelli.it (L.T.); 4 Gemelli-INTERACTS, Università Cattolica del Sacro Cuore, 00168 Roma, Italy; gyorgy.kovacs@unicatt.it

**Keywords:** interventional radiotherapy, vaginal-cuff brachytherapy, HDR brachytherapy, in vivo dosimetry, endometrial cancer, biological planning

## Abstract

(1) Background: Postoperative vaginal-cuff HDR interventional radiotherapy (brachytherapy) is a standard treatment in early-stage endometrial cancer. This study reports the effect of in vivo dosimetry-based biological planning for two different fractionation schedules on the treatment-related toxicities. (2) Methods: 121 patients were treated. Group A (82) received 21 Gy in three fractions. Group B (39) received 20 Gy in four fractions. The dose was prescribed at a 5 mm depth or to the applicator surface according to the distance between the applicator and the rectum. In vivo dosimetry measured the dose of the rectum and/or urinary bladder. With a high measured dose, the dose prescription was changed from a 5 mm depth to the applicator surface. (3) Results: The median age was 66 years with 58.8 months mean follow-up. The dose prescription was changed in 20.7% of group A and in 41% of group B. Most toxicities were grade 1–2. Acute urinary toxicities were significantly higher in group A. The rates of acute and late urinary toxicities were significantly higher with a mean bladder dose/fraction of >2.5 Gy and a total bladder dose of >7.5 Gy. One patient had a vaginal recurrence. (4) Conclusions: Both schedules have excellent local control and acceptable rates of toxicities. Using in vivo dosimetry-based biological planning yielded an acceptable dose to the bladder and rectum.

## 1. Introduction

In developed countries, endometrial cancer is the most common gynecological malignancy. In 2018, 382,069 new cases were estimated worldwide [1]. Most of the patients presented with an early-stage disease, where postoperative vaginal-cuff interventional radiotherapy (brachytherapy, VBT) was found to be non-inferior to postoperative external beam radiation treatment (EBRT) with equivalent rates of local vaginal recurrences as well as distant metastases [2,3].

Compared with EBRT or EBRT combined with VBT, VBT has better late toxicity sequences and offers a better quality of life. In addition to global health status, patients treated with VBT alone had a better social function [4,5,6,7,8]. Furthermore, the use of EBRT or EBRT combined with HDR-VBT resulted in higher treatment costs and higher toxicity without the survival benefit as compared to BT alone [9,10,11].

HDR-VBT as the sole adjuvant post-operative radiation technique is currently considered the standard care for many subgroups of patients with early-stage disease [12,13,14].

The American Brachytherapy Society (ABS) recommends treating the proximal 3–5 cm of the vaginal cuff with HDR-VBT postoperatively [3]. Different fractionation schedules are endorsed with the fraction size ranging between 2.5 and 7 Gy. The dose could be prescribed to the vaginal/applicator surface or at depth (3–5 mm). There is no optimal schedule, and the selection of fractionation schedules is usually dependent on the institutional experience and workload [3]. However, pathologic examination of the vaginal wall found that 95% of the lymphatic channels are located within a 3 mm depth of the vaginal surface. Subsequently, dose prescription to depth may provide adequate coverage of such lymphatics [15].

Nevertheless, prescribing the dose at a depth of 5 mm might subsequently result in increasing the dose to the nearby organs at risk (OAR), particularly the rectum and the urinary bladder, which in turn may increase the rates of treatment-related toxicities. Furthermore, changes in the patient’s anatomy and the applicator positioning differences between treatment fractions can lead to a change in the dose calculated to OAR [10].

In vivo dosimetry (IVD) measures the actual absorbed radiation dose in the patient while being treated. IVD is able to detect dose variation due to errors in dose calculation, applicator positioning errors, or changes in the patient’s anatomy [16].

At our institution, IVD is routinely used to measure the dose to OAR for the patients receiving postoperative HDR-VBT. The depth of dose prescription could be changed subsequently, hence biological planning. This retrospective study reports the effect of IVD-based biological planning for two different fractionation schedules on treatment-related toxicities.

## 2. Materials and Methods

### 2.1. Patients

Medical records of the patients with endometrial cancer who received postoperative adjuvant vaginal-cuff HDR-BT using IVD-based biological planning without EBRT from January 2006 to December 2014 were retrospectively reviewed. Only patients with a follow-up of at least 6 months were included in the analysis.

### 2.2. Vaginal Brachytherapy

From January 2006 to July 2010, the patients were treated with 21 Gy in 3 fractions over 3 weeks (group A). The active treatment length was the upper two-thirds of the rest-vaginal length. Afterwards, the treatment scheme was changed to 20 Gy in 4 fractions over 4 weeks (group B). Only the proximal 3 cm of the vaginal cuff was treated.

**Figure 1:**
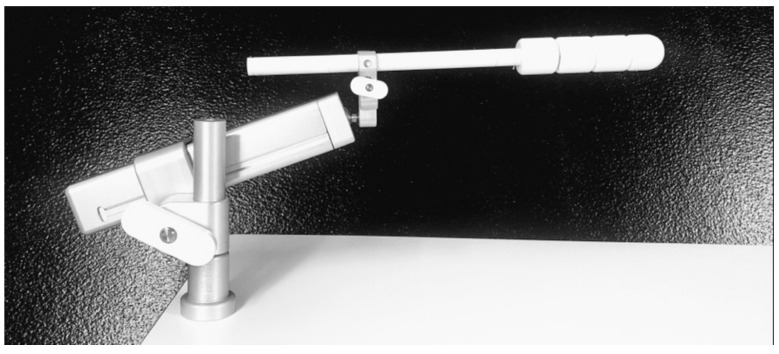
Cylinder vaginal applicator fixed using the fixing tray (Elekta Brachytherapy, Veenendaal, The Netherlands).

All the patients were treated using a vaginal cylinder applicator with the maximum diameter that could be introduced into the vagina (25, 30, and 35 mm). The applicator was introduced parallel to the treatment table and fixed using a fixing tray, Figure 1.

**Figure 2:**
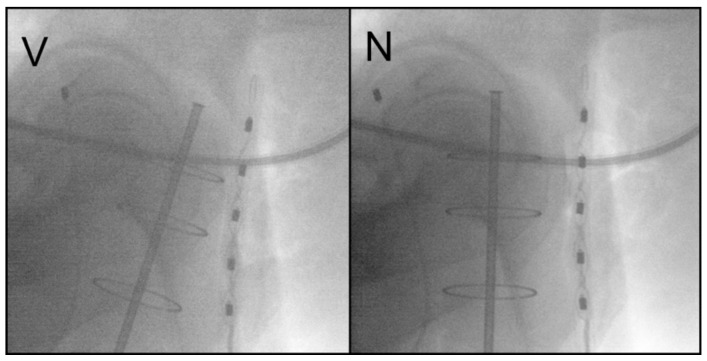
Lateral pre-interventional X-ray, before (V) and after (N) correction of the applicator position with consecutive reduction in the applied rectal dose.

Two orthogonal X-ray images were then acquired to verify the applicator position as well as the rectal and bladder points according to the international commission on reporting radiation units and measurements (ICRU), report number 38 [17]. Correction of the applicator position was performed when needed, Figure 2.

Generally, the dose was prescribed to a 5 mm depth of the applicator surface or to the applicator/vaginal surface. This was decided for each individual patient according to the measured distance between the applicator and the rectal probe on the pre-interventional orthogonal X-ray images. If the distance was below 0.5 cm, the dose was prescribed to the applicator/vaginal surface. Otherwise, the dose was prescribed to a 5 mm depth of the applicator surface.

### 2.3. In Vivo Dosimetry

A semiconductor-probes system was used to measure the dose to the rectum and/or urinary bladder (Multidos T10004; PTW, Freiburg, Germany). The rectal probe has five ionization chamber detectors; the highest measured dose of the five chambers was documented, while the bladder probe has only one measuring chamber.

IVD was used to measure the rectal and/or urinary bladder radiation dose. When the measured radiation dose during the first fraction was considered high, the dose prescription of the further fractions was changed from a 5 mm depth to the surface applicator. This led to a subsequent decrease in the measured rectal and/or urinary bladder radiation dose.

### 2.4. Follow-Up

Patients were regularly examined by a gynecologist every 3 months in the first 3 years, then every 6 months for 2 years. Pelvic and intravaginal ultrasound examinations were performed by the gynecologist, and additional investigations were carried out only if needed. A radiation oncologist documented the treatment-related acute toxicities under treatment as well as 6 weeks after treatment, and late toxicities were documented yearly. The grading of recorded toxicities was performed according to the Common Terminology Criteria for Adverse Events 4.0.

### 2.5. Statistical Analysis

Results are reported as the absolute value with the corresponding percentage, as the median with range, or as the mean with standard deviation (SD). A chi-square (χ2) test was used to compare categorical data and an independent T-test was used to compare continuous data. Follow-up duration was calculated starting from the date of HDR-BT to the date of death or last available visit. The statistical significance level was established at *p* < 0.05. Statistical analyses were performed using IBM SPSS Statistics for Windows, Version 20.0. (Armonk, NY, USA: IBM Corp).

## 3. Results

One hundred twenty-one patients were identified. The median age for the whole cohort was 66 years (44–87), and the mean follow-up period was 58.8 months (±33.4). The median interval between surgery and the start of brachytherapy was 6 weeks (range: 5–8). All patients had an early-stage disease (Stage I–II) except for only one patient who had a positive iliac lymph node where the EBRT was contraindicated due to wound complications. Patients and disease characteristics are summarized in Table 1.

Group A included 82 patients, while group B included 39 patients. In group A, the dose prescription was changed from 0.5 mm to the applicator/vaginal surface with a recorded mean rectal and/or bladder dose of 5.5 Gy (±0.9) in 20.7% of the patients. In group B, the dose prescription was changed with a mean dose of 2.6 Gy (±0.8) in 41% of the patients, Table 2.

In group A, the dose prescription was changed in 17 patients after the first fraction. In the second fraction, the mean rectal dose was reduced by a mean of 1.2 Gy (±1), and the mean urinary bladder dose was reduced by a mean of 1.3 Gy (±1). In group B, the change in prescription was documented in 16 patients. Subsequently, the mean rectal dose was reduced by a mean of 0.6 Gy (±0.2) and the mean urinary bladder dose was reduced by a mean of 0.4 Gy (±0.2) in the following fraction.

Table 3 shows the rectal and bladder mean doses per fraction as well as the mean total dose for three subgroups of patients according to the dose prescription; to the vaginal surface, to a 5 mm depth, and when the prescription was changed following IVD.

### 3.1. Acute and Late Radiation Toxicities

All recorded acute toxicities were grade 1–2. There was no significant difference in the rates of GIT or vaginal toxicities between the two treatment groups. Acute urinary reactions were significantly higher in group A patients, 31.7% versus 12.8% (*p* = 0.02). There was no difference in the distribution of late radiation reactions between both treatment groups, Table 4. Nearly all chronic toxicities were grade 1–2 except for one patient who had grade 3 chronic radiation cystitis and three patients who had grade 3 vaginal dryness; all grade 3 toxicities were among group A patients.

Further analyses were performed considering the mean dose per fraction as well as the mean total dose to the rectum for the whole cohort and its impact on the rates of GIT and urinary toxicities. No significant difference could be proved in the rates of acute as well as late GIT toxicities for patients with a mean rectal dose per fraction of >3 Gy versus ≤3 Gy. Likewise, with a mean total rectal dose of >12 Gy versus ≤12 Gy. However, most of the recorded toxicities were found in patients with higher doses, Table 5.

On the contrary, the rates of acute and late urinary toxicities were significantly higher with a mean bladder dose per fraction of ≤2.5 Gy versus >2.5 Gy (19.6% versus 33.3%, *p* = 0.04, and 6.5% versus 15.6%, *p* = 0.04 respectively). Moreover, there were more late urinary toxicities with a total bladder dose of ≤7.5 Gy versus ˃7.5 Gy (6.6% versus 15.2, *p* = 0.04), Table 5.

### 3.2. Treatment Outcomes

Only one patient in the whole cohort had vaginal vault recurrence (Stage Ib endometrioid adenocarcinoma). Distant and/or pelvic metastases were diagnosed in four patients (two endometrioid adenocarcinoma, one undifferentiated carcinoma, one serous adenocarcinoma). The mean time for metastases was 16.7 months (±7.1). Death was recorded in six patients with a mean survival of 46.2 months (±30.8). There was no difference in mean survival between patients in group A versus group B (44.7 months versus 47.8 months, *p* = 0.9)

## 4. Discussion

This study reports the clinical outcomes and the treatment-related toxicity following postoperative adjuvant VBT for early-stage endometrial cancer in two fractionation schedules using IVD-based biological planning. Both treatment groups showed acceptable rates of treatment-related toxicities and were mostly grade 1–2, which is in agreement with the results of randomized studies that compared VBT to EBRT [2,4,5,6,18,19]. Sorbe and Smeds evaluated four different fractionation schedules for HDR-VBT with fraction size ranging from 4.5 to 9 Gy, with dose prescription at 1 cm. Their results show that increasing the fraction size or the treatment length was associated with treatment-related toxicities [20]. Another report of 157 patients adopted a fraction size of 4 Gy for six treatments and prescribed the dose to the vaginal surface; no ≥ grade 2 treatment-related toxicities were found [21]. Furthermore, due to the excellent rates of local control as well as the low toxicities associated with the VBT, studies have focused on reducing the number of fractions or the total treatment time [22,23,24]. In the present study, two fractionation schedules were compared. Only acute urinary reactions of grade 1–2 were associated with a larger fraction size of 7 Gy compared to 5 Gy. No difference was shown for other toxicities between the two schedules.

Two-dimensional (2D) dosimetry calculated from orthogonal X-ray films is still widely adopted, especially in large centers with high workloads. The introduction of three-dimensional (3D) planning based on computer tomography (CT) images is usually limited only to the first fraction. The quality assurance protocol for the international ongoing molecular-integrated risk-profile-guided adjuvant treatment of endometrial cancer (PORTEC-4a) necessitates 3D planning during at least one fraction [25]. The comparing of both planning techniques was performed, with a correlation of the ICRU rectal and bladder points with a dose to 2cc on 3D [26]. There were no significant bladder dose differences between both planning techniques. However, rectal doses were significantly variable [26]. Owing to logistic limitations, 3D planning cannot be performed with every VBT fraction. Therefore, biologic planning based on IVD offers the advantage of monitoring the dose to the OAR during every treatment fraction and plane changes may be applied when needed.

In order to reduce the toxicity following VBT, dose prescription should be individualized. Several factors were found to play a role: the thickness of the vaginal wall, the anatomical variations, and the size of the used vaginal cylinder applicator [27,28]. Furthermore, the vaginal surface dose was found to be variable along the treatment length. With a 5 mm depth dose prescription, it ranged from 81% to 172% versus a range from 90% to 106% with a surface dose prescription. Both ranges are comparable and offer an acceptable coverage of the target [28].

In the present study, the selection of prescription depth was dependent primarily on the distance between the applicator and the rectal probe. For a smaller distance, a dose prescription at the applicator/vaginal surface was selected. Otherwise, a 5 mm depth for the prescription was preferred. During the first fraction, the dose to rectum and/or bladder was monitored by IVD. When the monitored dose was considered high, the prescription was changed to the surface if it was originally prescribed to a 5 mm depth. This approach led to a subsequent decrease in the monitored doses during the following fractions. Through our innovative two-step protocol, comparable mean doses to the bladder and rectum were achieved within both fractionation schedules.

Cut-off values of the mean dose per fraction as well as the mean total dose to the rectum and bladder were analyzed. Higher rates of toxicities were observed with higher doses, and statistical significance was partially confirmed probably due to the limitation of there being a small number of events.

Few studies have used IVD during HDR-VBT. The aim of IVD was mostly for real-time dose-rate monitoring and dose verification for quality assurance purposes [29,30,31]. One study used IVD to measure the actual dose to the rectum during HDR-VBT and compared it to the rectal dose as determined by the treatment planning system (TPS); the mean dose discrepancy was 2.2 Gy [32]. Other studies compared the actual dose to the rectum with the dose from TPS during HDR brachytherapy for prostate and cervix cancers [33,34,35]. In the current study, we used the IVD exclusively for monitoring the doses to the OAR, and modification of the plan occurred accordingly.

Notably, there are considerable limitations to using such an IVD system. Firstly, there is interfractional variation in the positioning of the rectal probe, which may be reflected in the subsequent measured doses. Secondly, as the rectal probe has five measuring chambers, we had to consider only the highest measurement. If the highest measurement is at one of the end measuring chambers, there would be a possibility that a further higher dose is not measured and thus the maximum rectal dose is not recorded. The values used in this work refer to the highest measured doses within the available measurement chambers and are therefore not to be always considered as the absolute rectal maximum doses. However, as the relative differences are considered and evaluated, a directional trend could be achieved.

Both fractionation schedules offered an excellent local control, and only one patient (0.8%) had a vaginal vault recurrence. Other studies reported local recurrence rates ranging from 0% to 3% [2,3,7].

## 5. Conclusions

Based on our results, postoperative HDR-VBT is a safe adjuvant treatment for early-stage endometrial cancer with excellent local control and acceptable rates of treatment-related toxicities. Both fractionation schedules, 21 Gy in three fractions and 20 Gy in four fractions, are comparable. Considering the individual patient’s anatomical variation together with IVD-based biological planning, an acceptable dose to the bladder and rectum could be achieved. Further studies are needed to validate the role of IVD during HDR-VBT.

## Figures and Tables

**Table 1:** Patients and disease characteristics.

Characteristics		
Age	Median (range)	66 (44–87)
BMI	Mean (±SD)	29.9 (±7.8)
Menopause	Premenopause	8 (6.6%)
	Postmenopause	113 (93.4%)
Histology	Endometrioid adenocarcinoma	114 (94.3%)
	Serous adenocarcinoma	5 (4.1%)
	Clear cell carcinoma	1 (0.8%)
	Undifferentiated carcinoma	1 (0.8%)
Grading	Grade 1	47
	Grade 2	44
	Grade 3	30
Lymphovascular space invasion	No	105 (86.8%)
	Focal	16 (13.2%)
	Substantial	0 (0%)
Lymphadenectomy	Yes	100 (82.7%)
	No	21 (17.3%)
Myometrial invasion	No	24 (19.8%)
	Less than 50%	45 (37.2%)
	More than 50%	52 (43%)
T-stage	pT1a	69 (57%)
	pT1b	40 (33.1%)
	pT2	12 (9.9%)
N-stage	Nx	17 (14%)
	cN0	4 (3.3%)
	pN0	99 (81.8%)
	N1	1 (0.8%)
FIGO clinical stage	Stage IA	69 (57%)
	Stage IB	39 (32.2%)
	Stage II	12 (9.9%)
	Stage IIIc	1 (0.8%)

SD = standard deviation; FIGO = Fédération Internationale de Gynécologie et d’Obstétrique.

**Table 2:** Brachytherapy dose prescription among treatment groups.

	Group A (No. 82)	Group B (No. 39)	All Patients (No.121)
Dose prescribed to vaginal surface	54 (65.9%)	16 (41%)	70 (57.9%)
Dose prescribed to 5 mm depth	11 (13.4%)	7 (17.9%)	18 (14.9%)
Dose prescription changed	17 (20.7%)	16 (41%)	33 (27.3%)

**Table 3:** Mean rectal and bladder doses between treatment groups.

	Group A (No. 82)		Group B (No. 39)	
	Mean rectal dose/fraction	Mean rectal total dose	Mean rectal dose/fraction	Mean rectal total dose
Dose prescribed to vaginal surface	3.9 Gy (±0.5)	11.5 Gy (±1.6)	2.7 Gy (±0.5)	10.6 Gy (±2.1)
Dose prescribed to 5 mm depth	4.1 Gy (±0.4)	12.4 Gy (±1.1)	3.2 Gy (±0.5)	12.8 Gy (±2.1)
Dose prescription changed	4.3 Gy (±0.5)	12.9 Gy (±1.4)	2.2 Gy (±0.6)	9.7 Gy (±2.2)
	Mean bladder dose/fraction	Mean bladder total dose	Mean bladder dose/fraction	Mean bladder total dose
Dose prescribed to vaginal surface	2.6 Gy (±0.5)	7.6 Gy (±1.6)	1.9 Gy (±0.4)	7.9 Gy (±2.1)
Dose prescribed to 5 mm depth	2.9 Gy (±0.6)	8.8 Gy (±1.9)	2.3 Gy (±0.4)	9.2 Gy (±1.4)
Dose prescription changed	2.7 Gy (±0.6)	8.1 Gy (±1.6)	2.1 Gy (±0.5)	8.2 Gy (±1.9)

**Table 4:** Acute and late radiation toxicity between treatment groups.

		Group A (No. 82)	Group B (No. 39)	*p*
Acute	GIT	10 (12.2%)	4 (10.3%)	0.6
	Urinary	26 (31.7%)	5 (12.8%)	0.02
	Vaginal	15 (18.3%)	6 (15.4%)	0.8
Late	GIT	5 (6.1%)	2 (5.1%)	0.4
	Urinary	14 (17.1%)	2 (5.1%)	0.7
	Vaginal	15 (18.3%)	8 (20.5%)	0.6

**Table 5:** Rectal and bladder doses in relation to GIT and urinary toxicities.

	Mean Rectal Dose/Fraction			Mean Rectal Total Dose		
	≤3 Gy (no. = 28)	˃3 Gy (no. = 84)	*p*	≤12 Gy (no. = 65)	˃12 Gy (no. = 47)	*p*
Acute GIT toxicities	2 (7.1%)	10 (13.1%)	0.6	8 (12.3%)	7 (12.8%)	0.9
Late GIT toxicities	1 (3.5%)	6 (7.1)	NA	1 (4.6%)	4 (8.5%)	NA
	Mean bladder dose/fraction			Mean bladder total dose		
	≤2.5 Gy (no. = 46)	˃2.5 Gy (no. = 45)	*p*	≤7.5 Gy (no. = 45)	˃7.5 Gy (no. = 46)	*p*
Acute urinary toxicities	9 (19.6%)	15 (33.3%)	*0.04*	11 (24.4%)	13 (28.3%)	0.9
Late urinary toxicities	3 (6.5%)	7 (15.6%)	*0.04*	3 (6.6%)	7 (15.2%)	*0.04*

## Data Availability

Data available on request due to restrictions, e.g., privacy or ethical. The data presented in this study are available on request from the corresponding author. The data are not publicly available due to institutional policy.
